# pVAC-Seq: A genome-guided *in silico* approach to identifying tumor neoantigens

**DOI:** 10.1186/s13073-016-0264-5

**Published:** 2016-01-29

**Authors:** Jasreet Hundal, Beatriz M. Carreno, Allegra A. Petti, Gerald P. Linette, Obi L. Griffith, Elaine R. Mardis, Malachi Griffith

**Affiliations:** McDonnell Genome Institute, Washington University School of Medicine, St. Louis, MO USA; Department of Medicine, Division of Oncology, Washington University School of Medicine, St. Louis, MO USA; Department of Medicine, Division of Genomics and Bioinformatics, Washington University School of Medicine, St. Louis, MO USA; Department of Genetics, Washington University School of Medicine, St. Louis, MO USA; Siteman Cancer Center, Washington University School of Medicine, St. Louis, MO USA; Department of Molecular Microbiology, Washington University School of Medicine, St. Louis, MO USA

## Abstract

Cancer immunotherapy has gained significant momentum from recent clinical successes of checkpoint blockade inhibition. Massively parallel sequence analysis suggests a connection between mutational load and response to this class of therapy. Methods to identify which tumor-specific mutant peptides (neoantigens) can elicit anti-tumor T cell immunity are needed to improve predictions of checkpoint therapy response and to identify targets for vaccines and adoptive T cell therapies. Here, we present a flexible, streamlined computational workflow for identification of *p*ersonalized *V*ariant *A*ntigens by *C*ancer *Seq*uencing (pVAC-Seq) that integrates tumor mutation and expression data (DNA- and RNA-Seq). pVAC-Seq is available at https://github.com/griffithlab/pVAC-Seq.

**Electronic supplementary material:**

The online version of this article (doi:10.1186/s13073-016-0264-5) contains supplementary material, which is available to authorized users.

## Background

Boon *et al.* were the first to demonstrate that cancer-specific peptide/MHC class 1 complexes could be recognized by CD8+ T cells present in cancer patients [1]. Substantial evidence now suggests that anti-tumor T cells recognize tumor somatic mutations, translated as single amino acid substitutions, as ‘neoantigens’. These unique antigenic markers arise from numerous genetic changes, acquired somatically that are present exclusively in tumor (mutant) and not in normal (wild-type (WT)) cells [2]. Recent preclinical data indicate that these mutated proteins, upon processing and presentation in the context of MHC molecules expressed by antigen-presenting cells, can be recognized as ‘non-self’ by the immune system.

Our previous work in murine sarcoma models was one of the first demonstrations of how somatic cancer mutations could be identified from massively parallel sequencing, and when considered in the context of MHC binding affinity, can predict tumor specific neoantigens [3]. A subsequent study further demonstrated that these neoantigens were the same epitopes recognized by anti-PD1 and anti-CTLA4 checkpoint blockade therapies and that peptide vaccines comprising neoantigens could provide prophylactic effects [4]. Several other studies have also characterized these neoantigens as being derived from somatically mutated genes in mouse [5] as well as in humans [6–9], and have shown that they can be recognized by T cells.

While checkpoint blockade therapies have achieved tremendous success in the clinic, patient-specific vaccines still meet a clinical need in those patients that either do not respond, develop resistance, or cannot tolerate the associated side effects of checkpoint blockade drugs. The main paradigm behind the development of cancer vaccines rests on the assumption that if the immune system is stimulated to recognize neoantigens, it may be possible to elicit the selective destruction of tumor cells. Vaccines incorporate these neoantigen peptides with the aim of enhancing the immune system’s anti-tumor activity by selectively increasing the frequency of specific CD8+ T cells, and hence expanding the immune system’s ability to recognize and destroy cancerous cells. This process is dependent on the ability of these peptides to bind and be presented by HLA class I molecules, a critical step to inducing an immune response and activating CD8+ T cells [10].

As we move from vaccines targeting ‘shared’ tumor antigens to a more ‘personalized’ medicine approach, *in silico* strategies are needed to first identify, then determine which somatic alterations provide the optimal neoantigens for the vaccine design. Ideally, an optimal strategy would intake mutation calls from massively parallel sequencing data comparisons of tumor to normal DNA, identify the neoantigens in the context of the patient’s HLA alleles, and parse out a list of optimal peptides for downstream testing. At present, elements of this ideal strategy exist, but are not available as open source code to permit others to adopt these methods into cancer care strategies. This manuscript describes one such approach, and provides a link to open source code for end users.

For example, to optimize identification and selection of vaccine neoantigens, several *in silico* epitope binding prediction methods have been developed [11–15]. These methods employ various computational approaches such as Artificial Neural Networks (ANN) and Support Vector Machines (SVM) and are trained on binding to different HLA class I alleles to effectively identify putative T cell epitopes.

There are also existing software tools (IEDB [16], EpiBot [17], EpiToolKit [18]) that compile the results generated from individual epitope prediction algorithms to improve the prediction accuracy with consensus methods or a unified final ranking. The current implementation of EpiToolKit (v2.0) also has the added functionality of incorporating sequencing variants in its Galaxy-like epitope prediction workflow (via its Polymorphic Epitope Prediction plugin). However, it does not incorporate sequence read coverage or gene expression information available from massively parallel sequencing datasets, nor can it compare the binding affinity of the peptide in the normal sample (WT) versus the tumor (mutant). Another multi-step workflow Epi-Seq [19] uses only raw RNA-Seq tumor sample reads for variant calling and predicting tumor-specific expressed epitopes.

We report herein an open source method called pVAC-Seq that we developed to address the critical need for a workflow that assimilates and leverages massively parallel DNA and RNA sequencing data to systematically identify and shortlist candidate neoantigen peptides from a tumor’s mutational repertoire that could potentially be used in a personalized vaccine after immunological screening. This automated analysis offers the functionality to compare and differentiate the epitopes found in normal cells against the neoepitopes specifically present in tumor cells for use in personalized cancer vaccines, and the flexibility to work with any user-specified list of somatic variants. Preliminary versions of this pipeline were applied in mouse models of cancer to identify expressed mutations in cancer cells and characterize tumor-specific mutant peptides that drive T cell-mediated tumor rejection in mice with MCA-induced sarcomas [3, 4]. More recently, we used this pipeline in a proof-of-concept trial in melanoma patients, to identify the neoantigen peptides for use in dendritic cell-based personalized vaccines [20].

## Methods

**Fig. 1:**
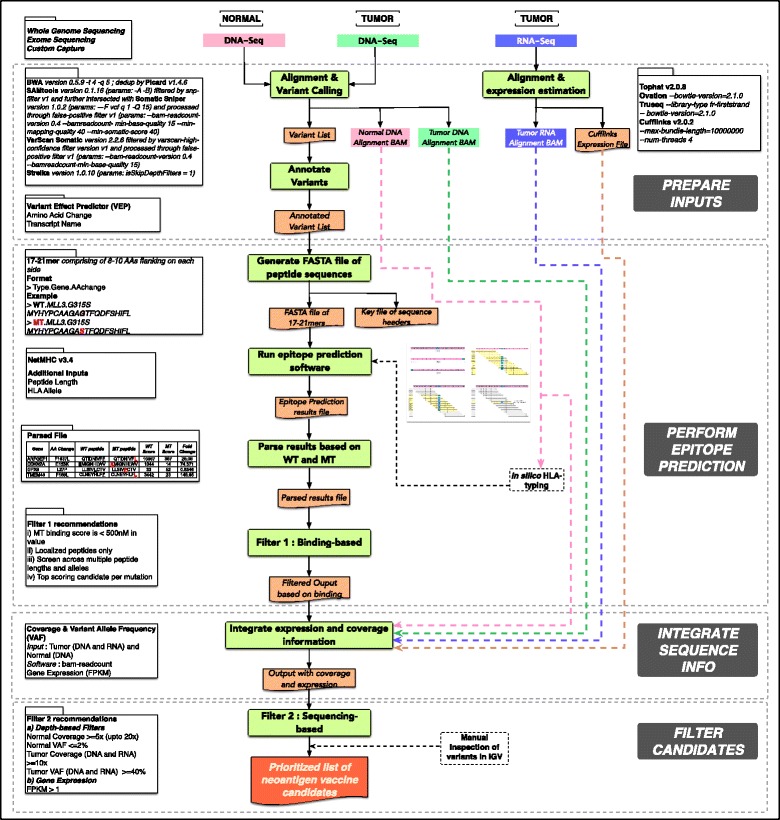
Overview of the pipeline pVAC-Seq: This figure illustrates the methodological framework behind the pVAC-Seq pipeline. Starting with preparation of inputs, it consists of three main steps - epitope prediction, integration of sequencing information, and filtered candidate selection

Our *in silico* automated pipeline for neoantigen prediction (pVAC-Seq) requires several types of data input from next-generation sequencing assays. First, the pVAC-Seq pipeline requires a list of non-synonymous mutations, identified by a somatic variant-calling pipeline. Second, this variant list must be annotated with amino acid changes and transcript sequences. Third, the pipeline requires the HLA haplotypes of the patient, which can be derived through clinical genotyping assays or *in silico* approaches. Having the above-mentioned required input data in-hand, pVAC-Seq implements three steps: performing epitope prediction, integrating sequencing-based information, and, lastly, filtering neoantigen candidates. The following paragraphs describe the analysis methodology from preparation of inputs to the selection of neoantigen vaccine candidates via pVAC-Seq (Fig. 1).

### Prepare input data: HLA typing, alignment, variant detection, and annotation

As described above, pVAC-Seq relies on input generated from the analysis of massively parallel sequencing data that includes annotated non-synonymous somatic variants that have been ‘translated’ into mutant amino acid changes, as well as patient-specific HLA alleles. Importantly, these data can be obtained from any appropriate variant calling, and annotation pipeline and HLA typing approach. Here, we outline our preparatory steps to generate these input data [20]. Somatic variant analysis of exome sequencing datasets was performed using the Genome Modeling System (GMS) [21] for alignment and variant calling. In brief, BWA (version 0.5.9) [22] was used for alignment with default parameters, except that the number of threads was set to 4 (-t 4) for faster processing, and the quality threshold for read trimming to 5 (-q 5). The resulting alignments were de-duplicated via Picard MarkDuplicates (version 1.46) [23].

In cases where clinically genotyped HLA haplotyping calls were not available, we used *in silico* HLA typing by HLAminer (version 1) [24] or by Athlates [25]. HLA typing was performed on the normal (peripheral blood mononuclear cells), rather than the tumor sample. Though the two software tools were >85% concordant in our test data (unpublished data), it is helpful to use both algorithms in order to break ties reported by HLAminer (see below).

I. HLAminer for *in silico* HLA-typing using Whole Genome Sequencing (WGS) data: When predicting HLA class I alleles from WGS data, we used HLAminer in *de novo* sequence alignment mode [26] by running the script HPTASRwgs_classI.sh, provided in the *HLAminer* download, with default parameters. (The download includes detailed instructions for customizing this script, and the scripts on which it depends, for the user’s computing environment.) For each of the three HLA loci, HLAminer reports predictions ranked in decreasing order by score, where ‘Prediction #1’ and ‘Prediction #2’ are the most likely alleles for a given locus. When ties were present for Prediction 1 or Prediction 2, we used all tied predictions for downstream neoepitope prediction. However, it should be noted that most epitope prediction algorithms, including NetMHC [13, 14], only work with an algorithm-specific subset of HLA alleles, so we are constrained to the set of NetMHC-compatible alleles. The current version NetMHC v3.4 supports 78 human alleles.
II. Athlates for *in silico* HLA-typing using exome sequence data: We diverged from the recommended Athlates protocol at two points: (1) We performed the alignment step, in which exome sequence data from the normal tissue sample are aligned against reference HLA allele sequences present in the IMGT/HLA database [27], using BWA with zero mismatches (*params : bwa aln -e 0 -o 0 -n 0*) instead of NovoAlign [28] with one mismatch. (2) In the subsequent step, sequence reads that matched, for example, any HLA-A sequence from the database were extracted from the alignment using bedtools [29] instead of Picard. This procedure is resource-intensive, and may require careful resource management. Athlates reports alleles that have a Hamming distance of at most 2 and meet several coverage requirements. Additionally, it reports ‘inferred allelic pairs’, which are identified by comparing each possible allelic pair to a longer list of candidate alleles using a Hamming distance-based score. We typically used the inferred allelic pair as input to subsequent steps in the neoepitope prediction pipeline.

After alignments (and optional HLA typing) were completed, somatic mutation detection was performed using the following series of steps (Additional file 1: Figure S1): (1) *Samtools* [30, 31] mpileup v0.1.16 was run with parameters ‘-A -B’ with default setting for the other parameters. These calls were filtered based on GMS ‘snp-filter v1’ and were retained if they met all of the following rules: (a) Site is greater than 10 bp from a predicted indel of quality 50 or greater; (b) The maximum mapping quality at the site is ≥40; (c) Fewer than three single-nucleotide variants (SNV) calls are present in a 10 bp window around the site; (d) The site is covered by at least three reads and less than 1 × 10^9^ reads; and (e) Consensus and SNP quality is ≥20. The filtered Samtools variant calls were intersected with those from *Somatic Sniper* [32] version 1.0.2 (params: -F vcf q 1 -Q 15), and were further processed through the GMS ‘false-positive filter v1’ (*params: --bam-readcount-version 0.4 --bamreadcount-min-base-quality 15 --min-mapping-quality 40 --min-somatic-score 40*). This filter used the following criteria for retaining variants: (a) ≥1% of variant allele support must come from reads sequenced on each strand; (b) variants must have ≥5% Variant Allele Fraction (VAF); (c) more than four reads must support the variant; (d) the average relative distance of the variant from the start/end of reads must be greater than 0.1; (e) the difference in mismatch quality sum between variant and reference reads must be less than 50; (f) the difference in mapping quality between variant and reference reads must be less than 30; (g) the difference in average supporting read length between variant and reference reads must be less than 25; (h) the average relative distance to the effective 3’ end of variant supporting reads must be at least 0.2; and (i) the variant must not be adjacent to five or more bases of the same nucleotide identity (for example, a homopolymer run of the same base). (2) *VarScan Somatic* version 2.2.6 [33, 34] was run with default parameters and the variant calls were filtered by GMS filter ‘*varscan-high-confidence filter version v1*’. The ‘*varscan-high-confidence v1*’ filter employed the following rules to filter out variants: (a) *P* value (reported by Varscan) is greater than 0.07; (b) Normal VAF is greater than 5%; (c) Tumor VAF is less than 10%; or (d) less than two reads support the variant. The remaining variant calls were then processed through false-positive filter v1 (*params: --bam-readcount-version 0.4 --bamreadcount-min-base-quality* 15) as described above. (3) *Strelka* version 1.0.10 [35] (*params: isSkipDepthFilters = 1*).

Our GMS pipeline expects a matched normal sample for filtering out potentially rare germline variants. However, in the absence of a matched normal tissue, the dbSNP and 1000 Genome databases could be used for filtering these variants.

The consolidated list of somatic mutations identified from these different variant-callers was then annotated using our internal annotator as part of the GMS pipeline. This annotator leverages the functionality of the Ensembl database [36] and Variant Effect Predictor (VEP) [37].

We wish to emphasize that any properly formatted list of annotated variants can be used as input to subsequent steps in the pipeline. From the annotated variants, there are two critical components that are needed for pVAC-Seq: amino acid change and transcript sequence. Even a single amino acid change in the transcript arising from missense mutations can alter the binding affinity of the resulting peptide with the HLA class I molecule and/or recognition by the T cell receptor. Larger insertions and deletions like those arising from frameshift and truncating mutations, splicing aberrations, gene fusions, and so on may also result in potential neoantigens. However, for this initial version of pVAC-Seq, we chose to focus our analysis on only missense mutations.

One of the key features of our pipeline is the ability to compare the differences between the tumor and the normal peptides in terms of the peptide binding affinity. Additionally, it leverages RNA-Seq data to incorporate isoform-level expression information and to quickly cull variants that are not expressed in the tumor. To easily integrate RNA-Seq data, both transcript ID as well as the entire WT transcript amino acid sequence is needed as part of the annotated variant file.

### Perform epitope prediction

**Fig. 2:**
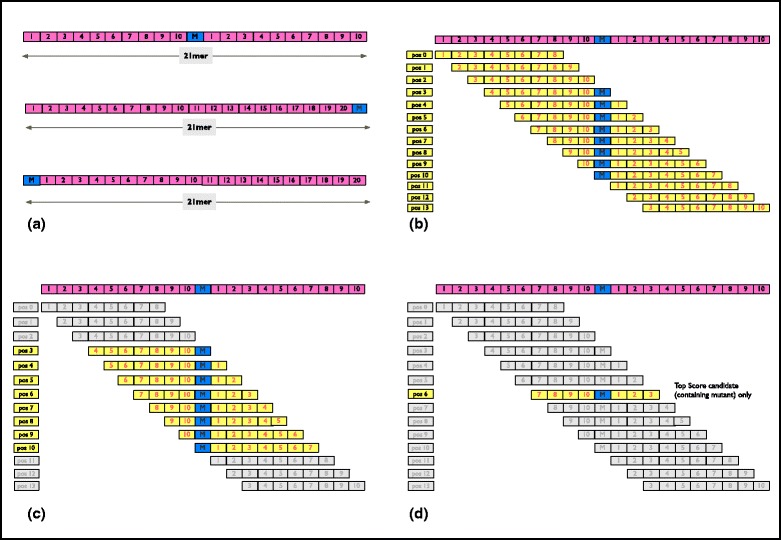
Generation of peptide sequences and filtering predicted epitope candidates. **a** Amino acid FASTA sequence is built using 10 flanking amino acids on each side of the mutated amino acid. The preceding or succeeding 20 amino acids are taken if the mutation lies near the end or beginning of the transcript, respectively. **b** All predicted candidate peptides from epitope prediction software based on selected k-mer window size. **c** Only localized peptides (those containing the mutant amino acid) are considered to compare to WT counterpart. **d** The ‘best candidate’ (lowest MT binding score) per mutation is chosen across all specified k-mers and between all independent HLA allele types that were used as input

One of the key components of pVAC-Seq is predicting epitopes that result from mutations by calculating their binding affinity against the HLA class I molecule. This process involves the following steps for effectively preparing the input data as well as parsing the output (Fig. 2).

#### Generate FASTA file of peptide sequences

Peptide sequences are a key input to the MHC binding prediction tool, and the existing process to efficiently compare the germline normal with the tumor is very onerous. To streamline the comparison, we first build a FASTA file that consists of two amino acid sequences per variant site: WT (normal) and mutant (tumor). The FASTA sequence is built using approximately eight to 10 flanking amino acids on each side of the mutated amino acid. However, if the mutation is towards the end or beginning of the transcript, then the preceding or succeeding 16 to 20 amino acids are taken, respectively, as needed, to build the FASTA sequence. Subsequently, a key file is created with the header (name and type of variant) and order of each FASTA sequence in the file. This is done to correlate the output with the name of the variant protein, as subsequent epitope prediction software strips off each FASTA header.

#### Run epitope prediction software

Previous studies [38, 39] have shown that allele-specific epitope prediction software, such as NetMHC, perform slightly better when compared to pan-specific methods such as NetMHCpan [40–42] in case of well-characterized alleles due to availability of large amounts of training data. However, pan-specific methods could be beneficial in cases where there is limited peptide binding data for training, for arbitrary HLA molecules, or when predicting epitopes for non-human species. We do anticipate adding this support for additional softwares in upcoming versions of pVAC-Seq. To predict high affinity peptides that bind to the HLA class I molecule, currently only the standalone version of NetMHC v3.4 is supported. The input to this software is the HLA class I haplotype of the patient, determined via genotyping or using *in silico* methods, as well as the FASTA file generated in the previous step comprising mutated and WT 17-21-mer sequences. Typically, antigenic epitopes presented by HLA class I molecules can vary in length and are in the range of eight to 11 amino acids (aa). Hence, we recommend specifying the same range when running epitope prediction software.

#### Parse and filter the output

Starting with the output list of all possible epitopes from the epitope prediction software, we apply specific filters to choose the best candidate mutant peptides. First, we restrict further consideration to strong- to intermediate-binding peptides by focusing on candidates with a mutant (MT) binding score of less than 500 nM. Second, epitope binding calls are evaluated only for those peptides that contain the mutant amino acid (localized peptides). This filter eliminates any WT peptides that may overlap between the two FASTA sequences. Our workflow enables screening across multiple lengths and multiple alleles very efficiently. If predictions are run to assess multiple epitope lengths (for example, 9-mer, 10-mer, and so on), and/or to evaluate all patient’s HLA-A, -B, and -C alleles, we review all localized peptides and choose the single best binding value representative across lengths (9 aa, 10 aa, and so on) based on lowest binding score for MT sequence. Furthermore, we choose the ‘best candidate’ (lowest MT binding score) per mutation between all independent HLA alleles that were used as input. Additionally, in the output file, the WT peptide binding score is provided. Although this score may not directly affect candidate choice or immunogenicity, end users may find this comparative information useful.

### Integrate expression and coverage information

We subsequently apply several filters to ensure we are predicting neoantigens that are expressed as RNA variants, and that have been predicted correctly based on coverage depth in the normal and tumor tissue datasets. We have found that gene expression levels from RNA-Seq data, measured as fragments per kilobase of exon per million reads mapped (FPKM), provide a good method to filter only the expressed transcripts. We used the tuxedo suite - Tophat [43, 44] and Cufflinks [45] - as part of the GMS to align RNA-Seq data and subsequently infer gene expression for our in-house sequencing data. Depending on the type of RNA prep kit, Ovation® RNA-Seq System V2 (NuGEN Technologies, Inc., San Carlos, CA, USA) or TruSeq Stranded Total RNA Sample Prep kit (Illumina, Inc., San Diego, CA, USA) used, Tophat was run with the following parameters: Tophat v2.0.8 ‘*--bowtie-version = 2.1.0*’ for Ovation, and ‘*--library-type fr-firststrand --bowtie-version = 2.1.0*’ for Truseq. For Ovation data, prior to alignment, paired 2 × 100bp sequence reads were trimmed with Flexbar version 2.21 [46] (*params: --adapter CTTTGTGTTTGA --adapter-trim-end LEFT --nono-length-dist --threads 4 --adapter-min-overlap 7 --maxuncalled 150 --min-readlength 25*) to remove single primer isothermal amplification adapter sequences. Expression levels (FPKM) were calculated with Cufflinks v2.0.2 (*params: --max-bundle-length = 10000000 --num-threads 4*).

For selecting unique vaccine candidates, targeting the best ‘quality’ mutations is an important factor for prioritizing peptides. Sequencing depth as well as the fraction of reads containing the variant allele (VAF) are used as criteria to filter or prioritize mutations. This information was added in our pipeline via *bam-readcount* [47]. Both tumor (from DNA as well as RNA) and normal coverage are calculated along with the VAF from corresponding DNA and RNA-Seq alignments.

### Filter neoepitope candidates

Since manufacturing antigenic peptides is one of the most expensive steps in vaccine development and efficacy depends on selection of the best neoantigens, we filter the list of predicted high binding peptides to the most highly confident set, primarily with expression and coverage based filters. The pVAC-Seq pipeline permits user-specified filters, and we encourage new users to experiment with these cutoffs in order to tailor the pipeline to their input data and analysis needs. We employ the following filters: (a) *Depth based filters*: We filter out any variants with normal coverage <=5× and normal VAF of >=2%. The normal coverage cutoff can be increased up to 20× to eliminate occasional misclassification of germline variants as somatic. Similarly, the normal VAF cutoff can be increased based on suspected level of contamination by tumor cells in the normal sample.

For tumor coverage from DNA and/or RNA, a cutoff is placed at >=10× with a VAF of >=40%. This ensures that neoantigens from the founder clone in the tumor are included, but the tumor VAF can be lowered to capture more variants, which are less likely to be present in all tumor cells. Alternatively, if the patients are selected based on a pre-existing disease-associated mutation such as BRAF V600E in the case of melanoma, the VAF of the specific presumed driver mutation can be used as a guide for assessing clonality of other mutations. Also, other known driver mutations such as KRAS G12/G13 or NRAS Q61 may be used to determine purity, and to subsequently adjust the VAF filters to target founder clone mutations. (b) *Expression based filters*: As a standard, genes with FPKM values greater than zero are considered to be expressed. We slightly increase this threshold to 1, to eliminate noise. Alternatively, we analyze the FPKM distribution (and the corresponding standard deviation) over the entire sample, to determine the sample-specific cutoffs for gene expression. Spike-in controls may also be added to the RNA-Seq experiment to assess quality of the sequencing library and to normalize gene expression data. Since alternative splicing can give rise to multiple transcripts that encompass the variant residue, optionally, all these transcripts could be included in analysis during the annotation step. However, one should be careful as this could potentially give rise to transcripts that do not include the variant. Also, long transcripts or transcripts with high G/C content might show some bias if RNA-CapSeq is used but in our experience are generally well represented. The primary goal of using RNA-(Cap)Seq data in our method is to address to questions of primary importance: (1) is the gene expressed at a reasonably high level (for example, FPKM >1); and (2) is the variant allele expressed in the RNA-seq fragment population.

This filtered list of mutations is manually reviewed via visual inspection of aligned reads in a genome viewer like IGV [48, 49] to reduce the retention of obvious false positive mutations.

### Dataset

To demonstrate the workings of our *in silico* pVAC-Seq pipeline, we applied it to four metastatic melanoma patients, the clinical results for three of whom were described previously [20]. In brief, there were three patients (MEL21, MEL38, MEL218) with stage III resected cutaneous melanoma, all of whom had received prior treatment with ipilimumab, and one patient (MEL69) with stage IV cutaneous melanoma. All four patients were enrolled in a phase 1 vaccine clinical trial (NCT00683670, BB-IND 13590) employing autologous, functionally mature, interleukin (IL)-12p70-producing dendritic cells (DC). Informed consent for genome sequencing and data sharing was obtained for all patients on a protocol approved by the Institutional Review Board of Washington University. We performed genomic analysis of their surgically excised tumors to select candidates for the personalized DC vaccine. Three of these patients (MEL21, MEL38, MEL69) had multiple metachronous tumors. Exome sequencing as well as RNA-CapSeq was performed for each of these tumors, and their corresponding matched normal tissue. The raw exome and transcriptome sequence data are available on the Sequence Read Archive database: Bioproject PRJNA278450, and corresponding dbGaP accession: phs001005.

## Results and Discussion

Since melanoma patients harbor hundreds of mutations, it can be challenging to filter down and target the best set of potentially immunogenic neoantigens for vaccine design. For each of the four metastatic melanoma patients, we used the annotated list of SNVs generated using the GMS strategy described above, and analyzed them via our pVAC-Seq pipeline. As mentioned earlier, for the demonstration of this workflow, amino acid changes resulting from only missense mutations were considered for analysis. Table 1 shows the breakdown of these SNVs described previously [20] and the data generated in subsequent steps through our workflow, leading to a high-confidence list of neoepitopes. As part of our local workflow, NetMHC v3.4 was used as the epitope prediction software to generate HLA class I restricted epitopes.

**Table 1:** Summary of predicted epitope candidates through pVAC-Seq pipeline

	MEL21			MEL38			MEL218	MEL69	
	LN	Skin	Skin	Axilla	Breast	AbWall	LN	Skin / Limb	Skin / Scalp
	(2011)	(2012)	(2013)	(2012)	(2013)	(2013)	(2005)	(2013)	(2013)
Total SNVs	702	838	1099	359	402	385	695	256	282
Missense SNVs	443	515	598	219	247	238	437	141	162
21-mer FASTA entries *(WT & MT)*	856	1,004	1,002	424	482	462	850	272	314
Raw NETMHC output *(9-mers)*	11,152*2 (HLA-A02:01, HLA-A01:01)	13,072*2 (HLA-A02:01, HLA-A01:01)	13,044*2 (HLA-A02:01, HLA-A01:01)	5,512*3 (HLA-A02:01, HLA-A31:01, HLA-B07:02)	6,270*3 (HLA-A02:01, HLA-A31:01, HLA-B07:02)	6,010 *3 (HLA-A02:01, HLA-A31:01, HLA-B07:02)	11,050*3 (HLA-A02:01, HLA-A03:01, HLA-B44:02	3,542*2 (HLA-A02:01, HLA-A11:01)	4,088*2 (HLA-A02:01, HLA-A11:01)
Parsed NetMHC output *(compared WT with MT)*	3,796*2 (HLA-A02:01, HLA-A01:01)	4,465*2 (HLA-A02:01, HLA-A01:01)	4,458*2 (HLA-A02:01, HLA-A01:01)	1,871*3 (HLA-A02:01, HLA-A31:01, HLA-B07:02)	2,131*3 (HLA-A02:01, HLA-A31:01, HLA-B07:02)	2,042*3 (HLA-A02:01, HLA-A31:01, HLA-B07:02)	3,770*3 (HLA-A02:01, HLA-A03:01, HLA-B44:02	1,217*2 (HLA-A02:01, HLA-A11:01)	1,395*2 (HLA-A02:01, HLA-A11:01)
**Filter 1:** Binding based	110	121	144	103	112	111	161	50	65
HLA-A02:01 candidates only	79	96	111	52	48	46	93	25	34
**Filter 2:** Manually reviewed HLA-A02:01 candidates (*Exome plus RNA-Seq*)	11	11	12	14	16	16	24	6	12
**Filter 3:** Experimentally tested	16			14			18	12	
**Filter 4:** Vaccine tested	7			7			7	10	
Immunogenicity	3			3			3	4	

The table illustrates the number of raw candidates predicted by NetMHC, and the parsing and filtering strategies applied thereafter to the final list of neoantigen candidates. These candidates were then communicated to our vaccine design collaborators who evaluated this list by patient-specific immunological assays (Filters 3 & 4) [20]

As is evident from Table 1, there were multitudes of epitopes reported by NetMHC v3.4 in its raw format. This number increased even further with the addition of each HLA class I allele. Using pVAC-Seq, and its recommended thresholds for filtering (binding and coverage-based), we were able to produce a more reasonable list of high affinity HLA class I binding neoantigen candidates for experimental validation.

**Fig. 3:**
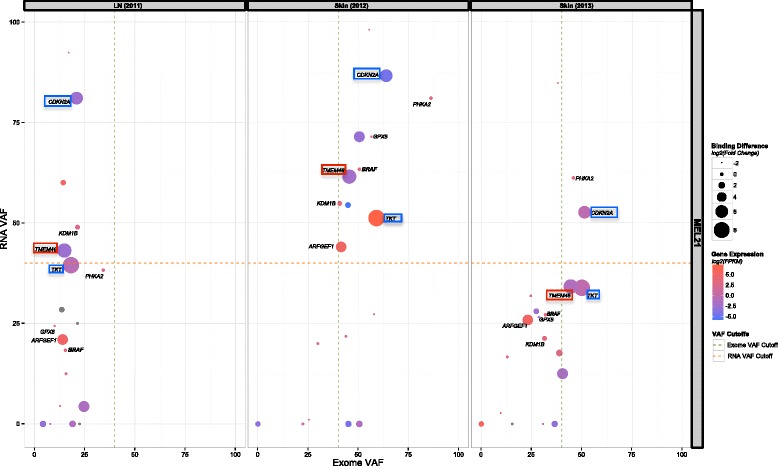
Landscape of filtered neoantigen candidates. This figure illustrates the landscape of neoantigen vaccine candidates in patient MEL21 after being prioritized using the pVAC-Seq pipeline. The points represent the overall sequencing information: exome and RNA VAFs, gene expression in terms of log2 FPKM value, as well log2 fold change, calculated as the ratio of WT binding affinity over mutant binding affinity. Recommended exome and RNA VAF cutoffs are also indicated. Candidates that were incorporated in the vaccine are labeled based on the genes containing these somatic mutations. Red boxes depict naturally occurring (that is, pre-existing T cell response) and blue boxes denote vaccine-induced neoantigens that were recognized by T cells. Since BRAF was used as a guide for assessing clonality of other mutations, it is also shown in each of three metachronous tumors (from the same patient)

These candidate neoantigens were experimentally tested in binding assays and those with confirmed binding to HLA class I restricting molecules were incorporated in the vaccine formulation [20]. Since all of these patients harbor the BRAF V600E mutation, we used its VAF in each sample as a comparative control of tumor purity and clonality. Integration of variant coverage information from Exome and RNA-Seq (VAF), as well as mutant expression information (FPKM), provided additional information needed to make an informed decision on the number and identity of peptides to include in each patient-specific vaccine (Fig. 3, Additional file 2: Figure S2, Additional file 3: Figure S3, and Additional file 4: Figure S4).

As shown, if existing epitope prediction software tools were solely used to generate neoantigen predictions in these patients, it would have been challenging to integrate the filters as well as the important digital sequencing metrics that ultimately determined the ‘quality’ of these candidates. By implementing the novel methods reported in this manuscript, we were able to rapidly streamline the screening and identification of a smaller number of potentially immunogenic neoepitopes within the landscape of all neoepitopes. This method can be further extended to include other genomic alterations such as frame-shift insertions and deletions, splicing aberrations, and gene fusions, which may in some cases cause larger changes in epitope binding affinities. We are currently testing approaches to include binding predictions from frame-shift insertions and deletions by incorporating VEP annotation, and once tested, will be adding this functionality to the github repository for pVAC-Seq. By expanding the focus from just somatic point mutations to the entire neoantigen landscape, it may also be possible to better assess whether neoantigen load itself can serve as a biomarker for prediction of checkpoint blockade response.

## Conclusions

The current regimen for predicting and screening neoantigens from sequencing data is laborious and involves a large number of intermediate steps such as creating FASTA files, running the prediction algorithms (most of the time online), and filtering output for high binding affinity candidates. Our flexible, automated *in silico* workflow, pVAC-Seq, provides higher efficiency and faster turnaround by automating many of these steps. This approach should help to evaluate tumor-specific neoepitopes in a much-reduced time, thereby increasing its applicability for clinical use. As we learn from ongoing early mouse and human trials, the methods developed will help optimize the composition of personalized cancer vaccines with high precision and will expedite vaccine design to address growing clinical demand.

## Additional files

Additional file 1: Figure S1. Illustrates the variant calling pipeline employed as part of the GMS strategy. (PDF 150 kb) Additional file 2: Figure S2. Illustrates the landscape of neoantigen vaccine candidates in patient MEL38 after being prioritized using the pVAC-Seq pipeline. The points represent the overall sequencing information: exome and RNA VAFs, gene expression in terms of log2 FPKM value, as well log2 fold change, calculated as the ratio of WT binding affinity over mutant binding affinity. Recommended exome and RNA VAF cutoffs are also indicated. Candidates that were incorporated in the vaccine are labeled based on the genes containing these somatic mutations. Red boxes depict naturally occurring (that is, pre-existing T cell response) and blue boxes denote vaccine-induced neoantigens that were recognized by T cells. Since BRAF was used as a guide for assessing clonality of other mutations, it is also shown in each of three metachronous tumors (from the same patient). (PDF 182 kb) Additional file 3: Figure S3. Illustrates the landscape of neoantigen vaccine candidates in patient MEL218 after being prioritized using the pVAC-Seq pipeline. The points represent the overall sequencing information: exome and RNA VAFs, gene expression in terms of log2 FPKM value, as well log2 fold change, calculated as the ratio of WT binding affinity over mutant binding affinity. Recommended exome and RNA VAF cutoffs are also indicated. Candidates that were incorporated in the vaccine are labeled based on the genes containing these somatic mutations. Red boxes depict naturally occurring (that is, pre-existing T cell response) and blue boxes denote vaccine-induced neoantigens that were recognized by T cells. Since BRAF was used as a guide for assessing clonality of other mutations, it is also shown. (PDF 111 kb) Additional file 4: Figure S4. Illustrates the landscape of neoantigen vaccine candidates in patient MEL69 after being prioritized using the pVAC-Seq pipeline. The points represent the overall sequencing information: exome and RNA VAF cutoffs, gene expression in terms of log2 FPKM value, as well log2 fold change, calculated as the ratio of WT binding affinity over mutant binding affinity. Recommended exome and RNA VAFs are also indicated. Candidates that were incorporated in the vaccine are labeled based on the genes containing these somatic mutations. Red boxes depict naturally occurring (that is, pre-existing T cell response) and blue boxes denote vaccine-induced neoantigens that were recognized by T cells. Since BRAF was used as a guide for assessing clonality of other mutations, it is also shown in both the metachronous tumors (from the same patient). (PDF 143 kb)
