# Supplementary figures and images for: pVAC-Seq: A genome-guided in silico approach to identifying tumor neoantigens
Source: Genome Med. 2016 Jan 29;8:11. doi: 10.1186/s13073-016-0264-5 (PMC4733280; doi:10.1186/s13073-016-0264-5)

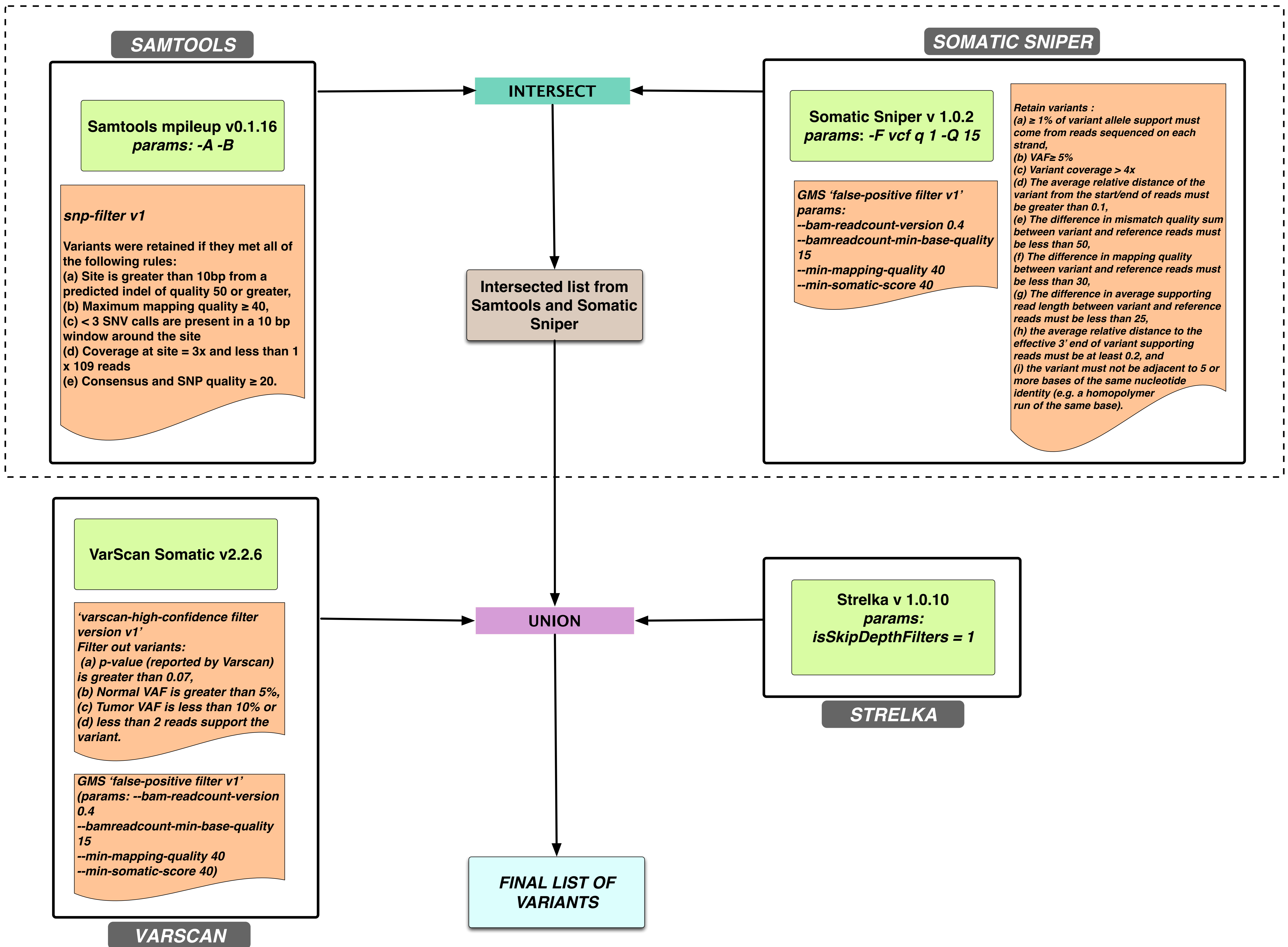

Supplement: Additional file 1: Figure S1. — Illustrates the variant calling pipeline employed as part of the GMS strategy. (PDF 150 kb) [file 13073_2016_264_MOESM1_ESM.pdf]

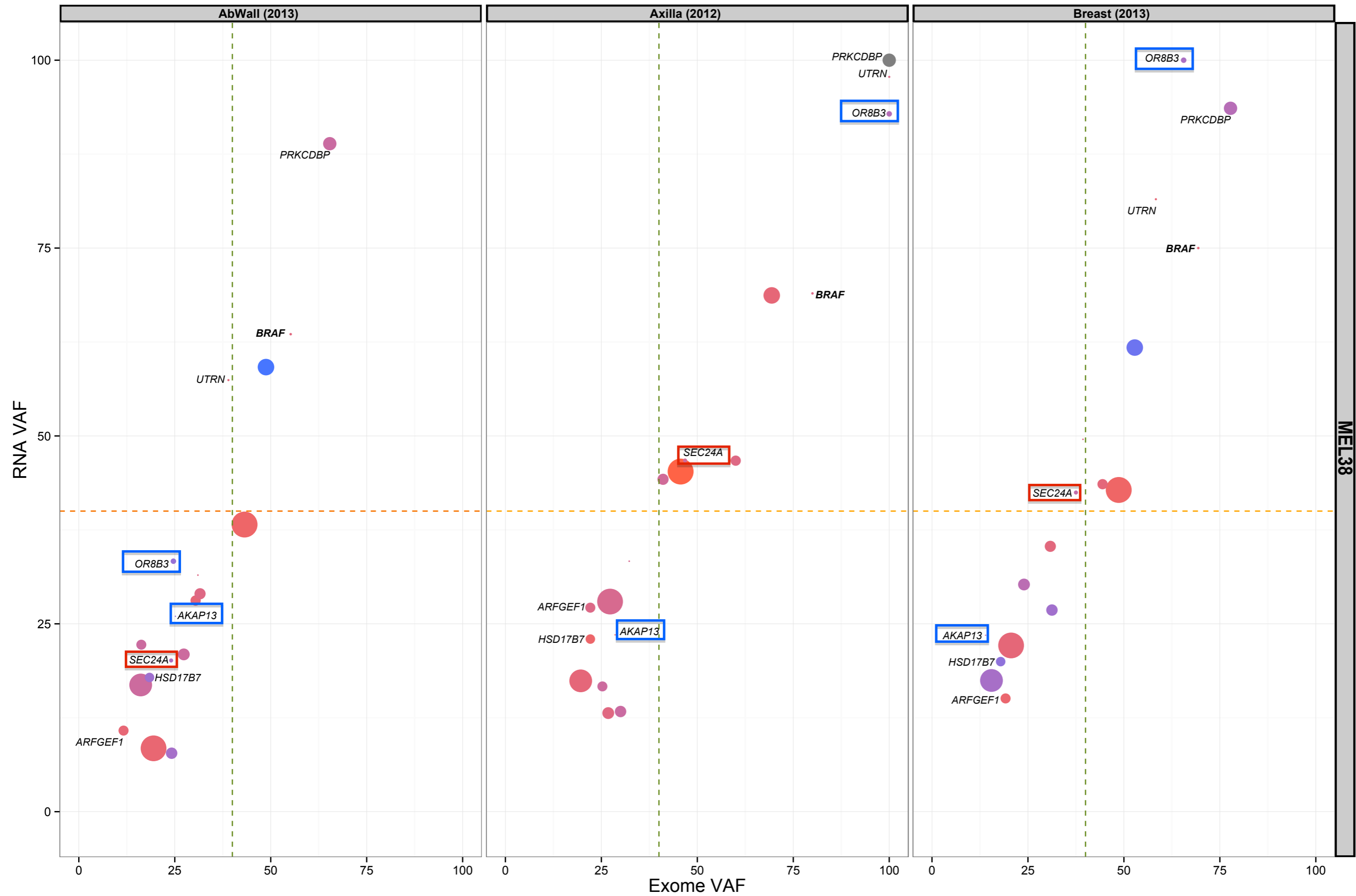

Supplement: Additional file 2: Figure S2. — Illustrates the landscape of neoantigen vaccine candidates in patient MEL38 after being prioritized using the pVAC-Seq pipeline. The points represent the overall sequencing information: exome and RNA VAFs, gene expression in terms of log2 FPKM value, as well log2 fold change, calculated as the ratio of WT binding affinity over mutant binding affinity. Recommended exome and RNA VAF cutoffs are also indicated. Candidates that were incorporated in the vaccine are labeled based on the genes containing these somatic mutations. Red boxes depict naturally occurring (that is, pre-existing T cell response) and blue boxes denote vaccine-induced neoantigens that were recognized by T cells. Since BRAF was used as a guide for assessing clonality of other mutations, it is also shown in each of three metachronous tumors (from the same patient). (PDF 182 kb) [file 13073_2016_264_MOESM2_ESM.pdf]

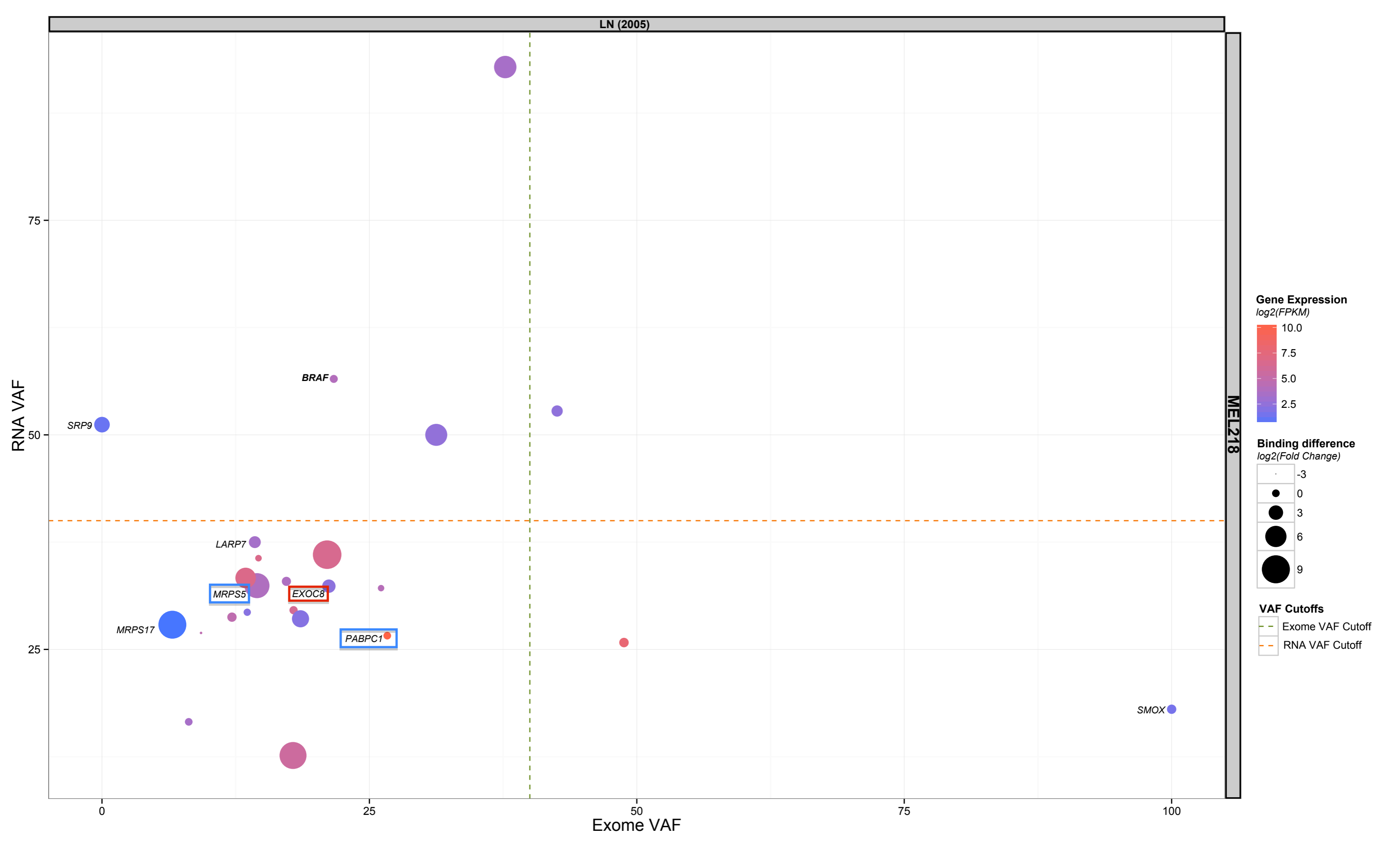

Supplement: Additional file 3: Figure S3. — Illustrates the landscape of neoantigen vaccine candidates in patient MEL218 after being prioritized using the pVAC-Seq pipeline. The points represent the overall sequencing information: exome and RNA VAFs, gene expression in terms of log2 FPKM value, as well log2 fold change, calculated as the ratio of WT binding affinity over mutant binding affinity. Recommended exome and RNA VAF cutoffs are also indicated. Candidates that were incorporated in the vaccine are labeled based on the genes containing these somatic mutations. Red boxes depict naturally occurring (that is, pre-existing T cell response) and blue boxes denote vaccine-induced neoantigens that were recognized by T cells. Since BRAF was used as a guide for assessing clonality of other mutations, it is also shown. (PDF 111 kb) [file 13073_2016_264_MOESM3_ESM.pdf]

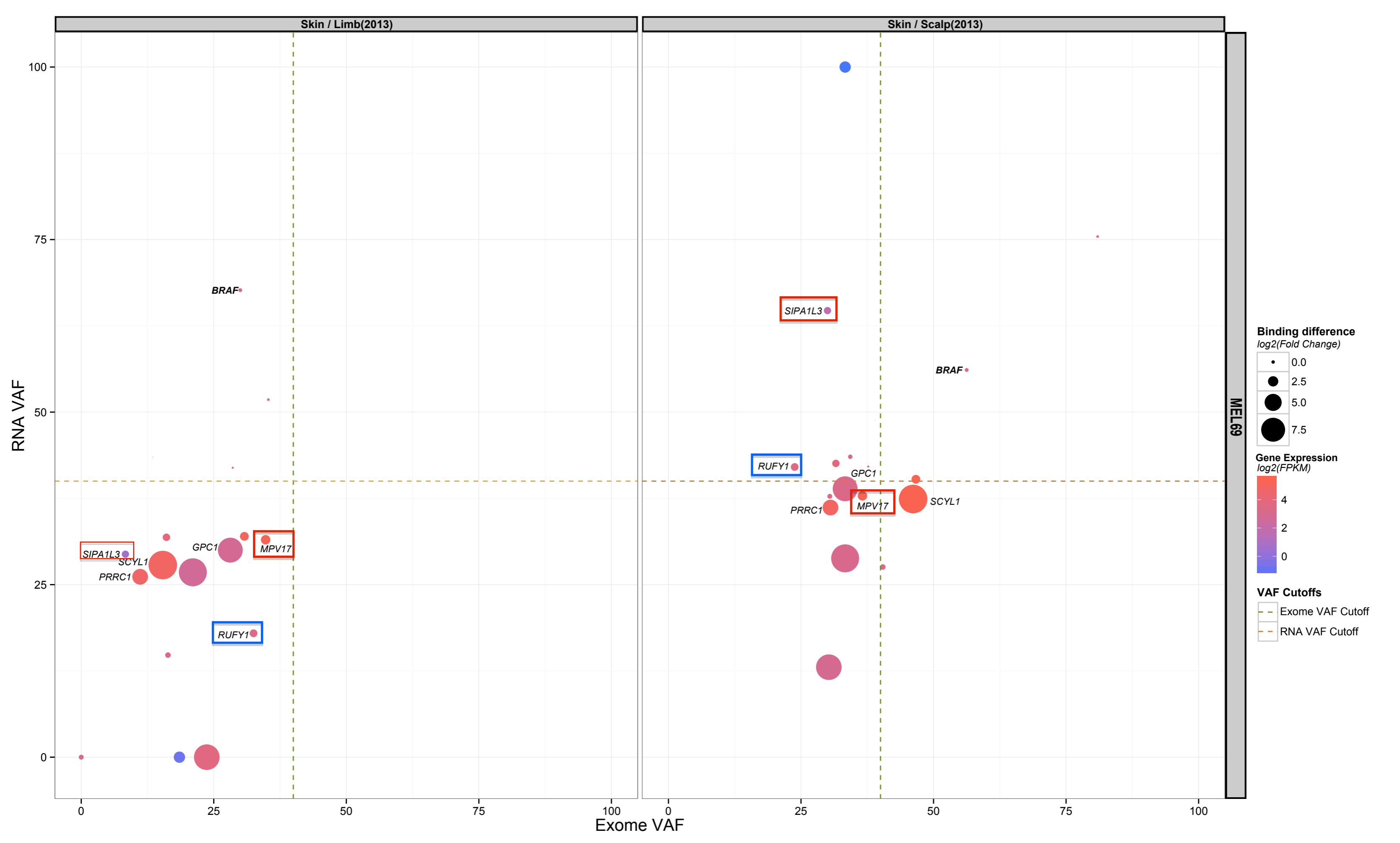

Supplement: Additional file 4: Figure S4. — Illustrates the landscape of neoantigen vaccine candidates in patient MEL69 after being prioritized using the pVAC-Seq pipeline. The points represent the overall sequencing information: exome and RNA VAF cutoffs, gene expression in terms of log2 FPKM value, as well log2 fold change, calculated as the ratio of WT binding affinity over mutant binding affinity. Recommended exome and RNA VAFs are also indicated. Candidates that were incorporated in the vaccine are labeled based on the genes containing these somatic mutations. Red boxes depict naturally occurring (that is, pre-existing T cell response) and blue boxes denote vaccine-induced neoantigens that were recognized by T cells. Since BRAF was used as a guide for assessing clonality of other mutations, it is also shown in both the metachronous tumors (from the same patient). (PDF 143 kb) [file 13073_2016_264_MOESM4_ESM.pdf]
